# There's Something about a Fair Split: Intentionality Moderates Context-Based Fairness Considerations in Social Decision-Making

**DOI:** 10.1371/journal.pone.0031491

**Published:** 2012-02-17

**Authors:** Sina Radke, Berna Güroğlu, Ellen R. A. de Bruijn

**Affiliations:** 1 Donders Institute for Brain, Cognition and Behaviour, Radboud University Nijmegen, Nijmegen, The Netherlands; 2 Institute of Psychology, Leiden University, Leiden, The Netherlands; 3 Leiden Institute for Brain and Cognition, Leiden University, Leiden, The Netherlands; Georgia State University, United States of America

## Abstract

Fairness considerations are a strong motivational force in social decision-making. Here, we investigated the role of intentionality in response to unfair offers in the Ultimatum Game by manipulating both proposers' degree of control over the selection of offers and the context pertaining to the outcomes of offers proposers can choose from. As a result, the design enabled us to disentangle intention- and context-based decision-making processes. Rejection rates were higher when an unfair offer was intentionally chosen over a fair alternative than when it was chosen by the computer, outside proposers' control. This finding provides direct evidence for intention-based decision-making. Also, rejection rates in general were sensitive to the context in which an offer was made, indicating the involvement of both intention- and context-based processes in social decision-making. Importantly, however, the current study highlights the role of intention-based fairness considerations in basic decision-making situations where outcomes are explicitly stated and thus easy to compare. Based on these results, we propose that fairness can be judged on different, but additive levels of (social-) cognitive processing that might have different developmental trajectories.

## Introduction

Fairness considerations, i.e. comparisons of self-interest and other-interest, are a strong motivational force in social decision-making. Individuals tend to regard outcomes that sustain normative expectations about fairness as most valuable [1]. Fairness norms prevail even in economic situations involving anonymous parties [2] and often imply a preference for an equal distribution of resources, i.e. inequity aversion [3], [4]. This process of social comparison is also assumed to be crucial for feelings of injustice, jealousy or envy [5], [6]. As many of our decisions are made within social settings, fairness intention models suggest that the intention of the interaction partner is crucial in fairness considerations [7]. Individuals show, for example, a greater desire to sanction intended unfair offers compared to unintended unfair offers in an economic game [8].

Perspective-taking is essential for evaluating others' intentions. Falk et al. [9] developed a modified version of the Ultimatum Game (UG) [10] to examine the role of intentionality in fairness considerations. In this version, the first player (proposer) chooses from a fixed set of two distributions of the stake (here 10 coins), which allows for manipulating the reference point of an offer. An unfair offer of 8 coins for the proposer and 2 coins for the responder (8∶2) is paired with four different alternatives: a fair- (5∶5), a hyperfair- (2∶8), a hyperunfair- (10∶0), and no-alternative (8∶2). Pairing an unfair offer (8∶2) with a fair alternative (5∶5) can be seen as an explicit version of the classic UG in which decision-making is usually driven by comparing an offer to a potential equal split, although this fairness norm remains implicit in the design. Previous studies using the modified UG paradigm have revealed that responders' behavior is sensitive to the alternative options. Rejection rates are highest when there is a fair-alternative, but lowest when paired with a hyperunfair- or no-alternative [9], [11], [12]. This suggests that fairness is not only evaluated based on the actual distribution, but also with regard to the alternatives to a given offer [13], [14], [15]. The authors [9], [11], [12] attributed higher rejection rates of unfair offers in the fair-alternative condition than in the no-alternative condition to intentionality considerations and perspective-taking and termed this ‘context effect’ [11], [16]. Therefore, throughout this paper the term “context” will refer to the manipulation of alternative offers, i.e. the unselected alternatives to an unfair offer. Further, it was assumed that the no-alternative condition implies that proposers make an unintended unfair offer because the two identical distributions do not permit a real choice so that neither ‘good’ nor ‘bad’ intentions could be inferred.

Alternatively, however, the difference in rejection rates across conditions (i.e. context effect) can be ascribed to comparing outcomes for the self in the proposed offer and the alternative distribution. In other words, the rejection decision may be based on the comparison of the possible gain for oneself (i.e. 2 coins in the 8∶2-distribution) and the alternative, but by now unattainable gain, e.g. 5 coins in the fair-alternative condition. Therefore, distributional concerns may lie at the heart of the context effect, which would be a more parsimonious explanation for the rejection pattern previously reported. Varying the reference point of an offer, i.e. the unchosen alternative, and thereby eliciting changes in responder behavior, does not necessarily have to involve perspective-taking or intentionality considerations, but has often been taken as an indication of these higher-order social processes [9], [11], [12], [15], [17]. Brandts & Solà [14] highlight that attributing intentions essentially requires non-outcome information, i.e. information that surpass a simple comparison of outcomes, and Sandbu [13] even claims that the context effect as such “reveals nothing about intentions.” Put differently, rejecting an unfair offer because it was of less value than the alternative (i.e. “I can get 2 coins now, but I could have gotten 5 coins”) can be anchored in relatively straightforward outcome comparisons without considering other players' perspective and their intentions.

In fact, a crucial factor in attributing intentions is proposers' degree of control in making a choice [7]. Previous studies [18], [19], [20], [21] have usually tried to capture this aspect by employing computer conditions in which participants play economic games against a computer. However, these studies tend to overlook an important aspect: When playing the UG against a computer - an inanimate proposer neither emotionally nor monetarily [18]–[20] affected by participants' decisions - considering any potential other-interest becomes pointless. Effectively, instead of depriving human proposers of their control over making an offer, proposers are replaced by computers that neither possess any sensitivity for fairness norms nor any authentic interest for the outcome of the game. The social quality of the interaction missing, it is not surprising that lower rejection rates of unfair offers from computers than from human proposers are reported. Remarkably, the study by Blount [21] is the only one in an UG setting in which participants' decisions actually had consequences for proposers' payoff, irrespective of who was in control of making an offer (a random device, a neutral third party or the other players themselves). When participants had to indicate the lowest amount that they would accept, a lower benchmark was set for offers generated by a random device than for offers determined by the other players themselves, which resembles the results from studies using computer conditions [18]–[20].

Unfortunately, the classic UG used in these experiments [18]–[21] is - due to its lack of an explicit reference point - not a suitable design to capture the sensitivity to contextual fairness. Conversely, the modified version of the UG [9], [11], [12] allows for varying the context of an offer. The no-alternative condition even implies that proposers have no actual choice in making an offer as the two options are equally unfair. Yet, the two factors of control and context comparisons of possible gains are confounded in this condition as an unfair offer involves both no-control over the offer *and* identical outcomes in the actual and alternative offer. In none of the existing designs [9], [11], [12], [15], [17] intentionality is treated as a separate factor that provides information going beyond available and alternative payoffs. Although some authors have proposed the development of models that incorporate both intention- and outcome-based fairness considerations [15], [22], the majority of experiments in both psychology and economics has focused on manipulating *either* intentionality or the context in which an offer occurs, i.e. the reference point. Put differently, replacing human proposers with a computer in order to capture intentionality neglects the influence of social comparisons [18]–[20]. On the other hand, varying the alternatives to an offer is often assumed to imply intentionality [9], [11], [12], [15], [17]. Yet, the latter design does not experimentally disentangle intentionality from the context effects, i.e. information about potential outcomes. Notably, no single study exists which adequately, i.e. explicitly, manipulates both contextual fairness and intentionality in an UG setting. Using a closely related game, the Dictator Game, in which the responder remains passive without a choice to accept or reject [23], Houser & Xiao [24], for instance, manipulate intentionality as well as context to investigate punishment behavior. Their results show that punishment is motivated by both inequality of the allocation and intentionality of the dictator, but with increased punishment when dictators themselves, instead of a computer, split the stake. However, context is created in a rather dimensional approach as dictators are presented with five different alternatives, ranging from none to the dictator's total endowment, so that there is always a better or worse alternative. As this scope is likely to trigger different perceptions of relative (un-)fairness than the binary choice set of the modified UG, we remain cautious to directly compare these two different designs.

The current study therefore aimed to investigate the relative roles of intention- and context-based fairness considerations. To accomplish this, we manipulated both the degree of control, i.e. intentionality, and context, i.e. the alternative offer, using a modified UG. Along with the context manipulations discussed above, we also included no-control conditions in which the computer takes over from the other player and randomly selects one of the two options. Consequently, conditions were established where the absolute payoff of a particular offer is the same, but it is either selected by the human player (control condition) or by the computer (no-control condition). For the control condition, we expected to replicate the previously reported relation between rejection rates and context as determined by the alternatives. For offers outside of proposers' control, we formulated two possible hypotheses: If intentionality is the central determinant of responders' decisions, rejection rates for all no-control conditions should be similar, irrespective of context. Moreover, these rejection rates should not differ from the no-alternative control condition because the same basic tendency for inequity aversion is expected in all conditions where proposers have no real choice, i.e. both in the no-alternative control and in all no-control conditions. Alternatively, however, if fairness considerations based on the comparison of gains in the actual and alternative offer are a central determinant of responders' decisions, the pattern of rejection rates should be similar for control and no-control conditions.

## Methods

### Participants

Fifty subjects (25 male, 25 female) participated in the experiment (*M* age = 22.52 yrs, *SD* = 5.43). All participants gave written informed consent and the procedure was approved by the local ethics committee (Ethische Commissie Gedragswetenschappelijk Onderzoek of the Faculty of Social Sciences at the Radboud University Nijmegen, The Netherlands).

### Design

Participants played the role of the responder in a computerized version of the modified UG. There were two within-subject factors: Control and Context. Control had two levels based on *who* selects the offer for the proposer: the human player him/herself (control) or the computer (no-control). The factor of control captures thus whether an offer was intended or not. Context had four levels based on alternatives to an unfair distribution (8∶2): a fair-alternative (5∶5 vs. 8∶2), a hyperfair-alternative (2∶8 vs. 8∶2), a hyperunfair-alternative (10∶0 vs. 8∶2), and no-alternative (8∶2 vs. 8∶2). Hence, the factor context pertains to the alternative outcome that had not been chosen. The resulting 8 conditions were presented 16 times each (counterbalanced for proposers' gender and position of the unfair offer). As the no-alternative condition entails an 8∶2 offer for either alternative, an unfair offer (8∶2) was presented in 5 of the 8 conditions, equivalent to 80 trials. The three genuine alternative offers (i.e., 5∶5, 2∶8 or 10∶0) were selected on 48 trials, yielding 128 trials in total. Contrary to subjects' belief, all choices were computer-generated.

### Material

**Figure 1:**
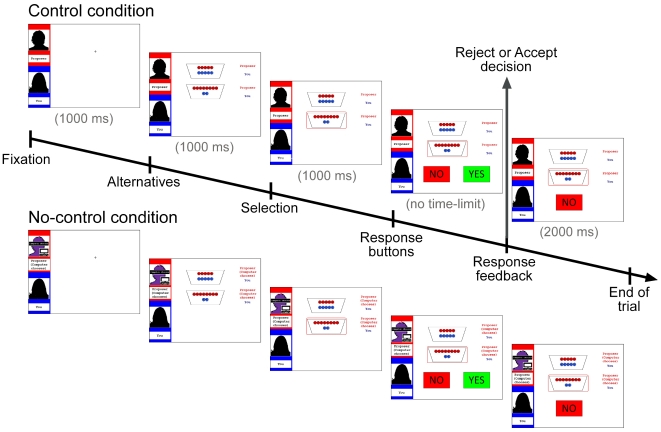
Display of a trial in the fair-alternative condition (top: control, bottom: no-control condition). The left part of the screen shows the name of the proposer at the top (here “Proposer”) and the name of the participant underneath (here “You”). In the no-control condition, the otherwise black silhouette of the proposer was purple with a banner displaying “Computer chooses”. The same banner was also displayed instead of the proposer's name. The two potential distributions are specified by red and blue coins (red for proposer, blue for responder). The offer selected by the proposer was encircled in red. The participant has to decide whether to accept (“Yes”) or reject (“No”) the offer via button press.

Figure 1 depicts the timeline of a trial in both fair-alternative conditions. Each round started with a fixation cross (1000 ms), followed by the presentation of the two available options (1000 ms). Next, the selected offer was surrounded by a red square (1000 ms). Subsequently, “Yes” and “No” buttons were presented while the selection remained visible. As the task was self-paced, participants had unlimited amount of time to respond via pressing one of two buttons using the keyboard. Participants' response remained on the screen for 2000 ms before the next round started.

### Procedure

Participants were led to believe that they were coupled with data from subjects who had previously participated as proposers and that they would play every round with a new partner. They were told that on some trials the other players would make an offer themselves and on other trials the computer would randomly select one of the two options. Participants' task was to decide whether to accept or reject an offer. If accepted, the coins were distributed as proposed; if rejected, neither player received anything. Participants were informed that at the end of the experiment, a random number of rounds would be selected to determine their payoff. This was done to assure participants' motivation and to strengthen the concept of a one-shot game as every round could influence their financial outcome. Moreover, it was emphasized that participants' decisions also affected the other players' outcome because their payoff would be determined by participants' response, irrespective of who made the proposal in a particular round (i.e. themselves vs. computer). Proposers would be paid after all data from responders had been collected. The payoff was set around 2.50 Euro to manage an equal payment for all participants, resulting in 10 Euro compensation.

## Results

**Figure 2:**
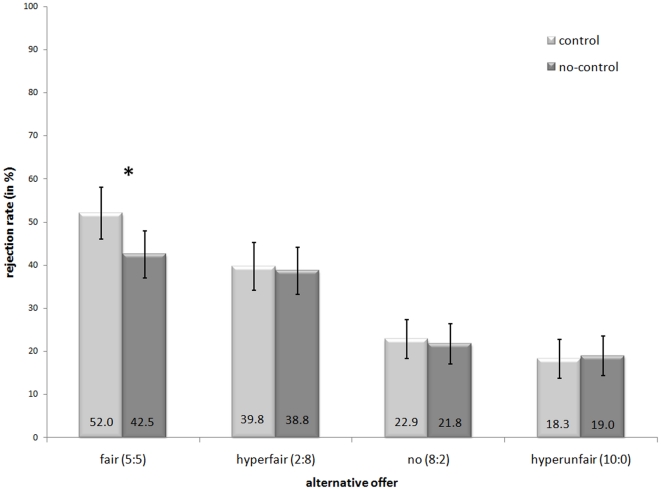
Rejection rates of unfair offers with regard to alternative offers and control of the choice. Mean percentage and standard errors of rejection of 8∶2-offers are displayed. Significant differences between control and no-control conditions are indicated by an asterisk(*), *p*<.01).

A repeated measures ANOVA was conducted for the rejection rate of unfair offers with control (two levels: human vs. computer) and context (four levels: fair vs. hyperfair vs. hyperunfair vs. no alternative) as within-subject factors. There was a main effect of control, *F* (1, 49) = 4.60, *p*<.05, *η^2^* = .09, indicating that rejection rates were highest when proposers' decisions were under their full control (33.2%) compared to when the computer took over and selected the offer (30.5%). Moreover, there was a main effect of context, *F* (3, 147) = 23.72, *p*<.001, *η^2^* = .33 (see Figure 2).

Post-hoc pairwise comparisons (using Bonferroni correction) revealed that rejection rates were highest for the fair-alternative condition (47.3%) compared to the other alternatives (hyperfair: 39.3%, *p*<.05; hyperunfair: 18.6%, *p*<.001; no-alternative: 22.3%, *p*<.001). Rejection rates for the hyperfair alternative condition were higher than for the hyperunfair and no-alternative condition (both *p*s<.001). The latter two did not differ significantly (*p* = 1).

Importantly, the interaction between context and control was significant, *F* (3, 147) = 5.80, *p*<.01, *η^2^* = .11. Further analyses demonstrated that this was due to the fair-alternative condition. Rejection rates were significantly higher in this condition when the proposal was made under proposers' full control (52.0%) than when the computer selected the offer (42.5%; *t*(49) = 3.10, *p*<.01).

For the alternative offers, i.e. for trials on which not the 8∶2, but the alternative distribution was chosen, hyperunfair offers were nearly always rejected (87.1%), whereas fair (5.1%) as well as hyperfair offers (2.5%) were nearly always accepted. This shows that the presence of basic fairness evaluations among participants can be assumed. There were no sex differences (all *p*s>.48).

## Discussion

The aim of the current study was to investigate the relative roles of intention- and context-based fairness considerations that drive social decision-making. Based on influential factors identified in earlier research [7], we manipulated (1) control, i.e. intentionality, as assessed by whether proposers themselves or the computer selected the offer, and (2) context, i.e., the outcome of the alternative in the modified UG.

An effect of control was evident when an unfair treatment was made explicit, namely when an unfair offer was paired with a fair alternative. Faced with a fair alternative, participants rejected unfair offers more often when the offer was selected by proposers themselves than when selected by the computer. In this specific situation, proposers' intentional deviation from the social norm of fairness was most salient. This finding thus provides direct evidence for intention-based decision-making processes in fairness considerations. When presuming that the situation in the classic UG most resembles the fair-alternative condition in the current design, our findings are technically in line with previous studies [18]–[21] reporting lower rejection rates of unfair offers from computers than from human proposers. However, one should bear in mind that the classic UG lacks an explicit reference point and is therefore not able to capture context effects and their potential interplay with other factors. Conversely, when playing the UG against a computer, responders' decisions usually do not have consequences for another player [18]–[20].

Responder behavior in conditions where proposers have full control over choosing an offer are in line with earlier studies investigating context effects [9], [11], [12]. Unfair offers were more often rejected when the alternative was fair compared to a hyperfair-, hyperunfair- or no-alternative. Moreover, the no-control conditions depicted a similar pattern of results as the control conditions, indicating that unfair offers were more often rejected when paired with a better (i.e. fair- or hyperfair-) alternative, even when the decision was not under proposers' control and clearly made unintentionally.

Taken together, the current results show that fairness considerations are sensitive to both intentionality and context. Comparing outcomes of the actual and the alternative offer remains an integral part of fairness evaluations even when the offers are beyond proposers' control. Importantly, however, intentionality becomes crucial when the unfair treatment is obvious: When proposers clearly choose *not* to offer an equal split by favoring an unfair distribution, the intentional social norm violation is instantly and unambiguously recognizable. Participants' increased tendency to reject these unfair offers can be regarded as a form of altruistic punishment – punishing proposers for the norm violation at a cost to themselves [25].

Previous studies have shown that altruistic punishment occurs frequently even in one-shot encounters, hence in absence of direct reciprocity or reputation formation [26]. It may be triggered by negative emotions like anger [20], [27] and is often used in order to reduce inequality [28], [29]. The current study shows that when confronted with two alternatives where one is strictly fair and the other undoubtedly unfair, the desire for compliance with fairness norms is most evident as participants were least willing to accept an intentional unfair offer. These results corroborate a study by Nelissen et al. [30] in which responders had an outside option that yielded a larger personal payoff when rejecting than when accepting an equal split. The majority of responders decided on behalf of an equal split, thus missing an additional monetary benefit. The preference for fairness norms overrules the motive of maximizing personal payoff, verifying that fairness is a normal good [4]. Despite a greater punishment tendency in response to intentional unfairness, altruistic punishment also occurs when the source of inequality is random, i.e. unintentional [8], [24], supporting the importance of egalitarian motives [28].

Although the strict and mutually exclusive differentiation between intention-based and outcome-based fairness seems to prevail in the literature [7], [29], [30], [31], [32], some authors have already expressed the need for models that include both intention- and outcome-based fairness considerations [15], [22]. Since our data suggest a broader perspective on fairness dimensions, we would like to propose a new framework that integrates key factors identified previously [7] as well as (social-)cognitive demands that accompany each level of fairness considerations.

In this new framework, outcome-based fairness considerations comprise the first and basic level of social decision-making. It is anchored in social comparison processes, that is, a comparison of outcomes of the self and the other. On this level, the concept of fairness primarily refers to an equal split, which is a very salient signal. A preference for an equal split - also referred to as inequity aversion - is already observable in young children [33], [34] and non-human species like capuchins, chimpanzees and domesticated dogs [35]. Experimental paradigms investigating this level usually involve the division of resources, as in the UG. Note that the context effects observed here and previously [9], [11]–[14], [17] exceed pure inequity aversion. As the magnitude of an offer, i.e. its absolute payoff for the self and the other, remains identical across contexts and degrees of control, objective fairness is violated in all unfair offers.

Taking into account the context in which an offer occurs characterizes the next level of fairness considerations. Here, additional information about the unchosen alternative has to be processed and integrated. This requires counterfactual thinking, i.e. mental representations of alternatives to past events [36] that, in this case, involve both the self and the other. Thinking about hypothetical events has been linked to executive functioning, especially working memory and inhibitory control [37]. Apart from engaging additional cognitive competencies, this level is likely to also pose more demands on mentalizing skills as chimpanzees and children do not show context effects in a modified UG [11], [12], [38]. The sensitivity to contextual fairness seems to go in hand with maturation in adolescence and seems to play an increasingly significant role in the decision-making process. Yet, the phenomenon of inequity aversion does not utterly disappear as rejection rates in the no-alternative conditions do not drop to zero, but remain substantial [9], [11], [12]. Hence, the rejection rate in the no-alternative conditions might reflect a basic tendency for inequity aversion [9], [17] that continues to exert influence on decision-making.

Intentionality can be viewed as the next level in fairness considerations. Judging whether observed behavior was intentional is crucial in social interactions as it may lead to different perceptions of responsibility and morality [39], [40]. In criminal law, for instance, intentionality comprises, apart from the voluntary element, also a cognitive facet, namely whether someone was aware of the probable consequences of an action. It is therefore often linked with outcome information and not regarded in isolation, as fairness intention models seem to suggest [7]. Our results support the notion that intentionality does not completely override the conclusions derived from previous levels as both the preference for equitable outcomes, i.e. a basic tendency for inequity aversion, as well as the sensitivity to contextual fairness remain. When an unfair treatment is explicit and salient, intentionality moderates context-based preferences. For future studies, it would be interesting to assess developmental changes that might complement the shift towards this level of decision-making.

These additive levels correspond to an increasing amount of information that is considered and integrated before a decision is made. Note that - unlike more complex everyday life situations - in the current task, all potential consequences are explicitly stated and can easily be compared using the same ‘currency’ (i.e. coins). In contrast, decoding and understanding intentions in real-life interactions is usually more complex and requires additional abilities, such as perspective-taking or mentalizing processes [41], [42] known to continue developing in humans until late adolescence [11]. The development of these different factors throughout adolescence is intriguing and should be explored in more detail.

Further research should be devoted to altered social decision-making that is frequently observed in clinical populations. Impairments in social decision-making have been associated with ventromedial prefrontal cortex (vmPFC) malfunction in e.g. lesion patients and patients with psychopathy or depression [43], [44], [45] and might be linked with specific processing deficits at one of these levels of fairness considerations. The use of sensitive methods such as eye-tracking could provide insight in the fixation patterns of both patient and healthy populations, e.g. the amount of time spent looking at the actual vs. the alternative offer and how this might be modulated by proposers' intention or other salient features. The inclusion of additional conditions with, for instance, equivalent absolute earnings, but differences in inequality (e.g. 2 coins for the responder being paired with 2 or 4 coins for the proposer respectively) would be highly relevant in order to get a better grip on the underlying mechanisms in future studies.

To conclude, the current study was the first to show that intentionality moderates context-based preferences when an unfair treatment is explicit and salient. However, outcome comparisons of the options that had initially been available shape fairness considerations even when unfairness was unintentional. Based on these results, we propose that fairness can be judged on different levels, which might have separate developmental trajectories. Whereas focusing on salient outcomes might be a useful initial rule of thumb, incorporating context-related information as well as correctly attributing intentionality is necessary for the complex social decision-making humans encounter in everyday life. In such real-life situations, information about all possible outcomes is not only sparse, but also highly complex and uncertain, yielding elevated fairness considerations to be efficiently driven by intention-based decision- making.
